# P23 A case of visceral leishmaniasis presenting as seropositive erosive rheumatoid arthritis in an immunocompromised patient

**DOI:** 10.1093/rap/rkae117.054

**Published:** 2024-11-05

**Authors:** Aqeel Maqsood Anjum, Jonathan Underwood, Celia Beynon

**Affiliations:** University Hospital of Wales, Cardiff, United Kingdom; University Hospital of Wales, Cardiff, United Kingdom; University Hospital of Wales, Cardiff, United Kingdom

## Abstract

**Introduction:**

Visceral leishmaniasis (VL) is a potentially fatal parasitic infection occurring primarily in the tropics, subtropics, and southern Europe. A strong humoral response can be observed in VL due to polyclonal activation of B-lymphocytes, and it can also result in the production of rheumatoid factor (RF), anti-CCP and anti-nuclear antibodies. We present this case here of visceral leishmaniasis presenting as seropositive RA on the background of HIV and hepatitis B infection.

**Case description:**

We present a case of a 46-year-old Caucasian male with a two-year history of skin nodules, symmetrical arthralgia and weight loss. He was diagnosed with HIV a decade ago which was well controlled with bictegravir, emtricitabine and tenofovir alafenamide. He received pulmonary tuberculosis (TB) treatment in 2012 and had chronic e-antigen-positive hepatitis B. He previously resided in Ukraine and travelled frequently to Crimea and the Middle East.

There was no history of Raynaud’s, chest pain, shortness of breath, sicca symptoms, mouth ulcers, genital ulcers, hair loss or photosensitivity.

On examination, he was cachectic with generalised lymphadenopathy and multiple subcutaneous (1-2cm, firm, mildly tender, non-ulcerating) forearm nodules. There was synovitis on two joints but no tenosynovitis or joint deformity. Initial investigations revealed  pancytopenia, hyperuricemia, proteinuria, positive RF (>200 IU/ml) and anti-CCP, positive ANA and anti-Ro antibody and erosion of proximal phalanx of the big toe bilaterally. His CD4 count was 166 (33%) with undetectable HIV viral load. Imaging showed lymphadenopathy and splenomegaly (17.5cm).

A diagnosis of rheumatoid arthritis with possible connective tissue disease overlap was made and he was initiated on hydroxychloroquine.

Because of systemic symptoms and atypical skin nodules, further investigation was undertaken including lymph node, bone marrow and skin biopsies, all of which confirmed a diagnosis of leishmaniasis. Blood PCR was positive for *L. donovani* complex, and serological analysis showed direct agglutination test and k39 Ag positivity.

Treatment was initiated by the infectious diseases team initially with amphotericin B 40 mg/kg monotherapy, later on further amphotericin B 30 mg/kg and miltefisone. He responded well as evidenced by reduced splenomegaly, resolution of skin nodules and improvement in cytopenia. His joint symptoms also improved except for the recurrence of ankle pain for which he received intra-articular steroids.

**Discussion:**

It has been widely reported in literature that leishmaniasis can trigger autoimmune diseases such as rheumatoid arthritis and systemic lupus erythematosis. There have also been multiple studies confirming the presence of rheumatoid factor, anti-CCP and ANA in patients with leishmaniasis without any presence of symptomatic autoimmune disease. The mechanisms involved in the pathophysiology of autoantibody productions are not yet fully understood.

There is some evidence that these are related to activation of polyclonal B cells due to a decreased regulatory T cell activity. Another possible mechanism would be production of autoantigen in response to cross reaction between the parasite and the hoarse tissue antigens. There have been multiple case reports of leishmaniasis in patients with rheumatoid arthritis who were taking anti-TNF biologics. This represents the reactivation of leishmaniasis due to immunosuppression. There has been a long-known link between HIV infection and leishmaniasis.

Our patient was a known case of HIV. Interestingly, not only did he have significantly positive rheumatoid factor, anti-CCP, ANA and anti-Ro antibodies, but he also had active synovitis on his joints and even erosions were seen on the X-ray. He fulfilled the criteria for rheumatoid arthritis.

Our case was unique in the sense that this is a case of visceral leishmaniasis in an HIV patient who presented with rheumatoid arthritis. Skin nodules in this patient were appearing similar to rheumatoid nodules. Later on, the biopsy confirmed that these were due to leishmaniasis. It is also a challenge to manage RA in patients with HIV especially when have already an opportunistic infection like leishmaniasis. A high prevalence of hyperuricemia in HIV infection has been reported in multiple studies. Our case did have raised urate but his disease was not consistent with gout clinically.

**Key learning points:**

• This is a rare case showing seropositive erosive RA coexisting with VL in an HIV patient, underscoring the link between autoimmune diseases and chronic infections.  Chronic infections are always considered an important differential in inflammatory arthritis or connective tissue disease especially when they present with systemic features of fever, weight loss and organomegaly.

• Although this disease is considered rare in the Western hemisphere, in the diverse, multiethnic and immigrant UK population this should be considered an important differential diagnosis. Another key element in clenching these diagnoses is detailed travel history. Multidisciplinary approach is key in managing such difficult cases. Our case was managed jointly by the rheumatology, dermatology and infectious disease teams, and the national leishmaniasis multidisciplinary team from University College London Hospitals.

• It is important to do a holistic assessment for these patients and get help from other specialities especially in such challenging cases.

